# Comparative study of gene set enrichment methods

**DOI:** 10.1186/1471-2105-10-275

**Published:** 2009-09-02

**Authors:** Luca Abatangelo, Rosalia Maglietta, Angela Distaso, Annarita D'Addabbo, Teresa Maria Creanza, Sayan Mukherjee, Nicola Ancona

**Affiliations:** 1 Istituto di Studi sui Sistemi Intelligenti per l'Automazione, CNR, Via Amendola 122/D-I, Bari, Italy; 2 Institute for Genome Science and Policy, Duke University, Durham, NC, USA

## Abstract

**Background:**

The analysis of high-throughput gene expression data with respect to sets of genes rather than individual genes has many advantages. A variety of methods have been developed for assessing the enrichment of sets of genes with respect to differential expression. In this paper we provide a comparative study of four of these methods: Fisher's exact test, Gene Set Enrichment Analysis (GSEA), Random-Sets (RS), and Gene List Analysis with Prediction Accuracy (GLAPA). The first three methods use associative statistics, while the fourth uses predictive statistics. We first compare all four methods on simulated data sets to verify that Fisher's exact test is markedly worse than the other three approaches. We then validate the other three methods on seven real data sets with known genetic perturbations and then compare the methods on two cancer data sets where our a priori knowledge is limited.

**Results:**

The simulation study highlights that none of the three method outperforms all others consistently. GSEA and RS are able to detect weak signals of deregulation and they perform differently when genes in a gene set are both differentially up and down regulated. GLAPA is more conservative and large differences between the two phenotypes are required to allow the method to detect differential deregulation in gene sets. This is due to the fact that the enrichment statistic in GLAPA is prediction error which is a stronger criteria than classical two sample statistic as used in RS and GSEA. This was reflected in the analysis on real data sets as GSEA and RS were seen to be significant for particular gene sets while GLAPA was not, suggesting a small effect size. We find that the rank of gene set enrichment induced by GLAPA is more similar to RS than GSEA. More importantly, the rankings of the three methods share significant overlap.

**Conclusion:**

The three methods considered in our study recover relevant gene sets known to be deregulated in the experimental conditions and pathologies analyzed. There are differences between the three methods and GSEA seems to be more consistent in finding enriched gene sets, although no method uniformly dominates over all data sets. Our analysis highlights the deep difference existing between associative and predictive methods for detecting enrichment and the use of both to better interpret results of pathway analysis. We close with suggestions for users of gene set methods.

## Background

One of the major goals in oncology is determining biological markers associated to onset, differentiation and progression of tumors, which could be potential targets for therapies [1]. Traditionally this objective has been pursued by a) measuring the expression levels of thousands of genes simultaneously in two different phenotypic conditions, and b) identifying those genes that are differentially expressed between disease phenotypes. It is well known that such an approach has serious limitations: the obtained results are poorly reproducible in studies on the same disease carried out in different laboratories; moreover much of the information associated to genes weakly connected with the phenotype is lost due to the univariate statistics usually adopted in these studies [2].

A common approach in expression analysis to overcome some of these issues is to combine the expression data with functionally or structurally related gene sets and examine over or under representation of these genes [3] with respect to genes that are differentially expressed. The key application of this setting is to assay the deregulation of sets of genes that encode functional or structural annotations such as pathways or chromosomal regions with respect to disease state. In this paper we use the terms enriched and deregulated gene set interchangeably to indicate gene sets statistically associated to the phenotype. A variety of methods have been developed for assessing the enrichment of sets of genes with respect to differential expression between two phenotypes or experimental conditions [2-9].

In this paper we present an empirical study to compare four of the above methods for assaying gene set enrichment. The methods we selected are Fisher's exact (FE) test [3], Gene Set Enrichment Analysis (GSEA) [2], Random-Set Methods (RS) [8] and Gene List Analysis with Prediction Accuracy (GLAPA) [7]. These approaches are representative of two distinct classes of methods to assess deregulation of gene sets. The first three methods use *associative statistics *and aim to quantify the deregulation of a gene set by measuring differences between the distributions of the expression levels of the genes belonging to the gene set in the two phenotypic conditions assayed. The criteria for selecting these particular methods were FE is the oldest method, GSEA is one of the most commonly used methods, and RS is computationally one of the most efficient methods. The fourth method uses a *predictive statistic *and quantifies the deregulation of a gene set by measuring the prediction accuracy of the phenotype of new subjects by using the expression levels of the genes in the gene set. GLAPA is the only predictive method in the above list.

The comparison of these four methods was carried out on simulated and real expression data. A simulation study was conducted in which we measured the ability of the methods to detect deregulated gene sets in which the deregulation is known by design. Moreover, we analyzed the accuracy of these methods on real data where we have strong a priori knowledge of which pathways or gene sets we expect to be differentially enriched between phenotypic conditions. This requirement is satisfied a) by studies where a model system is genetically perturbed and a gene set is defined as genes that most differentially express under the perturbation, as well as b) by expression studies where the pathways driving the phenotypic distinction are known. We have collected nine data sets that satisfy this requirement: five data sets with controlled genetic perturbations used to generate oncogenic signatures [10], two NCI-60 data sets where the phenotypic annotation strongly suggests which pathways should be differentially expressed, and data sets of breast and lung cancer [11,12] where our prior knowledge is weaker and limited.

We find that the performance of FE test is strongly influenced by the level of the test adopted to find differentially expressed genes. This method is the least sensitive and is shown to lack power. For these reasons it was excluded from the successive analysis. The other three methods, even though with substantial differences, are accurate and recover relevant gene sets. The simulation study highlights that no method outperforms all others consistently. In particular, GSEA and RS, in order, are able to detect weak one-sided deregulations. On the contrary, when up and down-regulated genes belong to the same gene set RS performs better than GSEA due to the particular statistics adopted. GLAPA is more conservative and larger differences between the two phenotypes are required to allow the method to detect deregulation of a gene set. The properties of the methods highlighted by the simulation study are confirmed by the analysis of the methods on real data sets. The activity of important oncogenes and pathways known to be deregulated in the experimental conditions and pathologies analyzed are detected although with different accuracy across the data sets. We find the ranking of enrichment of gene sets induced by GLAPA and RS to be very similar while GSEA produces somewhat different rankings. The ranking induced by GSEA is more similar to RS than GLAPA. Overall the rankings of all three methods share significant overlap. The conservative nature of GLAPA emerges in the analysis on real data and is due to the fact that it is based on a predictive score.

In the discussion section we provide users of gene set methods some practical advice on how to interpret the results of gene set analysis based on the empirical study we have conducted.

## Methods

### Data sets

Two different sets of data were used in our study (see Table 1). The first set was relative to microarray gene expression data in which the activity of particular oncogenes or the deregulation of given pathways were known. In [10], human primary mammary epithelial cell cultures (HMECs) were used for studying *in vitro *pathways associated to the activation of Myc, Ras, E2F3, Src and *β*-catenin oncogenes. To this end, recombinant adenoviruses were used for expressing the activities of these oncogenes in an otherwise quiescent cell and RNA from multiple independent infections were collected for DNA microarray analysis using Affymetrix Human Genome U133 Plus 2.0 Array. Each experiment was composed of gene expression profiles of HMECs with activated oncogene and profiles of HMECs expressing green fluorescent protein, GFP, as control. Moreover we used a dataset with a known P53 perturbation from the NCI-60 collection of cancer cell lines, profiled by using Affymetrix Human Genome U95 Array (hgu95av2). This dataset included 12 normal samples and 50 samples with a P53 mutation. Finally, we considered an expression data set composed of 3 human astrocytes and 3 epithelial cells (HeLa cells) maintained under hypoxic conditions and 3 human astrocytes and 3 HeLa cells maintained under normal conditions [13], profiled by using Affymetrix Human Genome U133 Plus 2.0 Array. The second set of data was relative to microarray gene expression data of real human tumors. In [11], gene expression profiles were obtained for 60 individuals with hormone receptor-positive primary breast cancer treated with adjuvant tamoxifen monotherapy. Of these individuals, 32 experienced tumor recurrence. In [12], patients affected by non-small cell lung cancer (NSCLC) were profiled by using Affymetrix Human Genome U133 Plus 2.0 Array. The dataset was composed of 45 adenocarcinoma lung cancer samples and 48 squamous lung cancer samples.

**Table 1:** Data sets used in our experiments. The breast cancer data set is annotated by gene symbols.

**Dataset**	**Study**	**Class I vs Class II**	**# Probes**
Myc	[10]	10 vs 10	54675
Ras	[10]	10 vs 10	54675
E2F3	[10]	9 vs 10	54675
Src	[10]	7 vs 10	54675
*β*-catenin	[10]	9 vs 10	54675
P53	NCI-60	12 vs 50	12625
Hypoxia	[13]	6 vs 6	54675

Breast	[11]	28 vs 32	15017
Lung	[12]	45 vs 48	54675

All the data sets were properly normalized according to the procedure adopted in their original papers. In particular, oncogene [10], P53 and lung [12] data sets were normalized by using Robust Multiarray Average (RMA) procedure; Hypoxia data set [13] was normalized by using GCOS1.2 with the advanced PLIER (probe logarithmic intensity error) algorithm; breast data set [11] was normalized by using the robust nonlinear local regression method proposed in [14].

### Gene sets

The database of gene sets used in this paper was the Molecular Signatures Database (MSigDB) [2]. This is a collection composed of 1692 curated gene sets based on high-throughput experiments as well as expert knowledge from literature or databases. We added 10 gene sets to this database that were defined in [15]. To compare the three methods, we assessed the enrichment of all the gene sets in the experimental conditions and diseases examined.

### Algorithms

We are given a data set *S *= {(**x**_1_, *y*_1_), (**x**_2_, *y*_2_),..., (**x**_ℓ_, *y*_ℓ_)} composed of ℓ labelled specimens, where **x**_*i *_∈ ℝ^*d*^, *y*_*i *_∈ {-1, 1} for *i *= 1,2,...,ℓ and *d *is the number of probes on the microarray in the adopted technology. Let us suppose we have ℓ_+ _positive and ℓ_- _negative examples, such that ℓ = ℓ_+ _+ ℓ_-_. Moreover, we are given a gene set *G *= {*g*_1_, *g*_2_,..., *g*_*m*_} composed of *m *probes, where *m *≪ *d*.

#### RS

Let *s*_*i*_, *i *= 1,..., *d*, be a score associated to each probe. This score is a quantitative measure of differential expression which in our case is based on a two sample t-statistic for each gene *t*_*i*_, the two samples are the two phenotypes or conditions. Specifically, *s*_*i *_= |Φ^-1^((*t*_*i*_))|, *i *= 1,..., *d*, where *t*_*i *_were the two-sample t-statistics values computed for each gene, (*t*_*i*_) = *rank*(*t*_*i*_)/*d *where *rank*(*t*_*i*_) is the rank of the value *t*_*i *_in the array [*t*_1_,..., *t*_*d*_], and Φ is the standard normal cumulative distribution function. Given these scores the measure of gene set deregulation is *Z *= ( - *μ*)/*σ*, where  is the average of gene scores, , and *μ *= ℰ{} and *σ *= var{} are easily computed from the full set of gene scores.

Large values of *Z *are expected if *G *is deregulated in the experimental conditions analyzed. *P*-values are computed using phenotypic permutation test [16] and false discovery rate (FDR) computations are provided using the method described in [4].

#### GLAPA

This method uses an estimate of the generalization error of predictors trained by using raw expression levels of the genes belonging to *G *as a measure of enrichment of *G *[7]. Unbiased estimates of the generalization error were obtained through multiple cross validation strategies [17]. To this end, we build a reduced data set  composed of ℓ examples consisting only of probes corresponding to the genes in *G*. The cross validation is implemented by randomly splitting  into a pair (, ) of training and test sets with *h *and *k *examples respectively, ℓ = *h *+ *k*. A linear classifier is trained using the examples in  and its error rate *e*_*i *_was evaluated by testing the classifier on . The random splitting of  was repeated 200 times and the error rate *e*_*G *_associated to *G *was evaluated as the average of *e*_*i*_, *i *= 1,..., *s*. The assessment of the statistical significance of the measured *e*_*G *_was carried out by two independent permutation tests.

The first test (T1) controls for how likely the error rate *e*_*G *_was due to chance and we performed 1000 random permutations of the phenotypic label to compute this p-value. The second permutation test (T2) controls for the effect of the gene set size in the error rate *e*_*G *_and is performed by randomly selecting gene sets of the same size as *G *and recomputing *e*_*G*_. We used 1000 random gene sets to compute this p-value. The FDR in each permutation test was estimated with the method described in [4].

#### GSEA

This method uses a variation of a Kolmogorov-Smirnov statistic to provide an enrichment score for each gene set. Although numerous and more sophisticated variants of this method exist (see for example [18]), we refer to the original work of Subramanian [2]. This version of the methodology uses a variation of rank statistics where the ranks are weighted by the absolute value of the association of gene expression with phenotype, the weighting is added to overcome the granularity of rank based methods - there is a loss of sensitivity. As in the random set method a score measuring the correlation of a probe with the phenotype is required, *s*_*i*_, *i *= 1,..., *d*. We use the signal-to-noise metric in the standard GSEA setting as our score.

This metric is very similar to the two sample t-statistic used in our implementation of RS. Based on these correlation scores and the adjusted Kolmogorov-Smirnov statistic we compute an enrichment score which is signed. The weighting parameter in the adjusted Kolmogorov-Smirnov statistic is the absolute value of the correlation statistic, this is also the default parameter in the distributed software. Negative scores correspond to down-regulation of the gene set and positive scores correspond to up-regulation of the gene set. These enrichment scores are then normalized to take into account the size of the gene sets resulting in a normalized enrichment score. This normalization is done based on phenotypic permutations followed by standardization, see [2]. P-values as well as false discovery rates are computed using the standard setting of the software.

## Simulation study

The performances of the various methods used in the paper were assessed through a simulation study in which the amount of deregulation and the number of differentially expressed (DE) genes in a gene set were known by design. To this end, we adopted the same scheme suggested in [9] and simulated 1000 genes and 50 samples in each of 2 classes, control and treatment. The genes were assigned to 50 gene sets, each with 20 genes. All measurements were generated as No(0,1) before the treatment effect was added. There were five different scenarios:

1. all 20 genes of gene set 1 are 0.2 units higher in class 2;

2. the 1st 15 genes of gene set 1 are 0.3 units higher in class 2;

3. the 1st 10 genes of gene set 1 are 0.4 units higher in class 2;

4. the 1st 5 genes of gene set 1 are 0.6 units higher in class 2;

5. the 1st 10 genes of gene set 1 are 0.4 units higher in class 2, and 2nd 10 genes of gene set 1 are 0.4 units lower in class 2.

In every scenario only the first gene set is of potential interest. For each scenario, we repeated 20 simulations and, for every simulation, we carried out 1000 permutations of the phenotypic labels to compute the p-value of RS and GSEA and the p-value1 of GLAPA, and we used 1000 random gene sets with 20 genes to compute the p-value2 of GLAPA. The mean and standard error of the p-values computed over the 20 simulations are reported in Table 2.

**Table 2:** Results of simulation study: comparison of RS, GSEA and GLAPA. P-values for the first gene set for the three methods (columns) and five different scenarios (rows) described in the text.

	**RS**		**GLAPA**				**GSEA**	
	**P-value**		**P-value1**		**P-value2**		**P-value**	
				
	**mean**	**se**	**mean**	**se**	**mean**	**se**	**mean**	**se**
1	0.0177	0.0069	0.0678	0.0135	0.0173	0.0057	0.0002	0.0001
2	0.0005	0.0002	0.0152	0.0036	0.0007	0.0003	0.0002	0.0001
3	0.0004	0.0002	0.0064	0.0026	0.0003	0.0002	0	0
4	0.0126	0.0081	0.0073	0.0025	0.0002	9e-05	0.0166	0.0044
5	0	0	0.0001	8e-05	0	0	0.0877	0.0151

We extended the simulations to study the effect of heavier tails and dependence between genes in the gene set. To model heavier tails we used the Student's t-distribution to generate the measurements. To model dependence between genes we used the normal distribution with strong positive covariance to generate measurements. Neither of these variations resulted in appreciable differences in the simulation results (see Table 1 and 2 in Additional file 1).

Unlike the other three methods a threshold is required to select a subset of significantly DE genes when using Fisher's exact test. We used a t-test with specified *α *to select the set of genes of which we measure the overlap with genes in the gene sets. The simulation results for various levels of *α *are presented in Table 3. Comparing the simulation results of Fisher's exact test versus the other three methods (see Table 2) illustrates the lack of power of this approach. This test is unable to detect gene sets with modest deregulation and its performance is strongly influenced by the level *α *adopted to find DE genes. For these reasons we excluded the Fisher's exact test in the comparisons in the results section.

**Table 3:** Results of simulation study: Fisher's exact test.

	***α *= 0.01**		***α *= 0.02**		***α *= 0.03**		***α *= 0.04**		***α *= 0.05**	
	**P-value**		**P-value**		**P-value**		**P-value**		**P-value**	
					
	mean	se	mean	se	mean	se	mean	se	mean	se
1	0.4117	0.0901	0.2789	0.0773	0.1509	0.0411	0.1243	0.0406	0.1137	0.0427
2	0.1961	0.0795	0.0342	0.0171	0.0270	0.0217	0.0287	0.0265	0.0140	0.0120
3	0.0085	0.0033	0.0019	0.0011	0.0024	0.0010	0.0034	0.0020	0.0053	0.0034
4	0.0030	0.0017	0.0016	0.0006	0.0039	0.0018	0.0081	0.0037	0.0113	0.0039
5	2e-04	0.0002	1e-06	0	1e-06	0	6e-07	0	7e-07	0

P-values for the first gene set in the five different scenarios (rows) described in the text. In each column we report the significance level (*α*) adopted in t-test to find DE genes.

The simulation study on the other three methods highlights that no method outperforms all others consistently. In particular, GSEA and RS are able to detect weak deregulations between control and treatment groups, as long as the percentage of DE genes in the gene set is greater than 50% as in the first three scenarios. Note that the performances of RS increase as the amount of deregulation of the gene set increases. Their performances decrease when only the 25% of the genes belonging to the gene set are DE as in the 4th scenario. Finally, as the 5th scenario shows, RS performs better than GSEA when a two-sided deregulation in opposite directions occurs in the same gene set. This property is due to the particular score function adopted in RS which uses the absolute value. On the contrary, the amount of deregulation strongly influences the performances of GLAPA. Large differences are required between the two groups to allow GLAPA to detect deregulation of a gene set. Moreover, differently from RS and GSEA, this method is poorly influenced by the percentage of DE genes in the gene set. In fact, as the 4th scenarios shows, GLAPA is able to detect the deregulation even whether only the 25% of the genes is DE in the gene set. This property is particularly relevant when we assess the statistical significance of the deregulation in the second permutation test T2 in which the error rate of the gene set is compared with the error rate of random gene sets with the same size. These two aspects highlight the conservative nature of this method.

## Results

Comparison of the three methods can be summarized in terms of three aspects: validation of the gene set methods, differences in gene set ranks across the methods, and differences due to associative versus predictive scores.

The measure of evidence of enrichment for a gene set is the *Z *score for RS, the absolute value of the normalized enrichment score (NES) for GSEA, and the cross-validation error *e*_*G *_in GLAPA.

### Validation of the three algorithms

For each of the gene sets we have some prior knowledge of which gene sets should be deregulated. For some of the data sets such as the P53, Hypoxia, and the five oncogenic perturbations we have very strong knowledge of which gene sets should be deregulated since the genetic perturbation is very controlled. In the lung cancer and breast cancer data there are many genetic perturbations and these are not controlled samples. However, due to prior biological knowledge we still have some weaker expectations of which gene sets should be deregulated.

For validating the three methods we define for each data set a core set composed of gene sets thought to be involved in biological or cellular processes relevant in a data set. The reason for considering the core set as a whole is that gene sets are constructed under a variety of contexts and conditions and looking at a group of sets helps average out this variation. In addition to providing evidence for the enrichment and significance of individual gene sets we provide a summary statistic of the enrichment of the core set as well as the significance of this summary. The summary we use in this paper is the median rank of the gene sets in the core set and we use a permutation procedure much like a sign-rank test to assess significance.

#### P53 perturbation data

The NCI-60 collection of cancer cell lines contains 50 samples with P53 mutation and 12 normal samples. We expect to find enrichment of gene sets corresponding to pathways associated with P53 mutation in this data set. P53 is a tumor suppressor gene involved in the apoptotic signaling circuitry. In particular, the P53 protein is a transcription factor that normally inhibits cell growth and stimulates cell death when induced by cellular stress [19]. The results of the three methods applied on the whole MSigDB gene set collection are reported in Additional file 2.

In MSigDB we found 12 gene sets associated at varying levels to P53 deregulation. These defined our core set, see Table 4. This core set is composed of P53 gene sets as well as P21, hypoxia, and BRCA1 gene sets. P21 is relevant since it is a downstream effector of P53 that mediates both G1 and G2/M phase arrest and may be induced during P53-mediated apoptosis [20]. BRCA1 is involved in p53-mediated growth suppression [21]. Hypoxic conditions elicit P53 overexpression and consequent apoptosis.

**Table 4:** Results for the P53 gene sets in the Wild-Type/P53 mutant data set.

		**RS**		**GLAPA**			**GSEA**	
				
**Pathway**	**Size**	**Rank**	**P-value**	**Rank**	**P-value1**	**P-value2**	**Rank**	**P-value**
**STRESS_P53_SPECIFIC_UP**	17	2	0.000	3	0.004	0.000	2	0.000
**P53GENES_ALL**	30	4	0.000	9	0.013	0.000	8	0.000
**P53PATHWAY**	41	10	0.000	10	0.019	0.000	5	0.000
**KANNAN_P53_UP**	52	9	0.001	11	0.018	0.000	6	0.000
**P53HYPOXIAPATHWAY**	38	21	0.001	15	0.022	0.000	4	0.000
**P53_BRCA1_UP**	40	137	0.068	66	0.084	0.008	1	0.000
P53_SIGNALING	163	59	0.023	60	0.075	0.000	203	0.063
P21_P53_EARLY_DN	14	75	0.103	619	0.348	0.329	47	0.017
P21_P53_ANY_DN	46	470	0.294	1212	0.548	0.736	66	0.067
P21_P53_LATE_DN	11	661	0.357	1218	0.527	0.650	454	0.323
P21_P53_MIDDLE_DN	21	1381	0.744	1243	0.544	0.683	440	0.307
KANNAN_P53_DN	25	1478	0.893	1015	0.476	0.569	944	0.548

As Table 4 shows collectively the core set is strongly deregulated with respect to P53 mutation. The median scores are 67, 63, and 27.5 for RS, GLAPA, GSEA respectively and these are all significant *p *< 0.001. We ordered the gene sets according to the mean rank over the three methods in Table 4 and found the top six (in bold) to be highly ranked across all methods with median scores for this subset of 9.5, 10.5, and 4.5 for RS, GLAPA, and GSEA. One observation is that when P53 signatures were split into up-regulated and down-regulated sub-signatures the down-regulated gene sets were not consistently enriched. This is clearly illustrated by comparing the KANNAN_P53_UP and KANNAN_P53_DN signatures. Indeed five of the gene sets with low or mixed ranks correspond to P53 sub-signatures of down-regulation.

In summary the three methods are consistent across the twelve core gene sets and six of these accurately represent P53 mutation status.

#### Hypoxia data

The hypoxia data set is composed of 6 samples under hypoxic conditions and 6 samples under normal conditions. Hypoxia refers to the condition a cell experiences under oxygen deficiency. In this conditions, numerous adaptive responses are activated at molecular and cellular level, including alteration of gene expression. Alternatively, cancer cells can genetically elicit a hypoxic response in the setting of normal oxygen levels to activate new blood vessel formation to experience a growth advantage. The results of the three methods applied on the whole MSigDB gene set collection are reported in Additional file 3. In MSigDB we found 19 gene sets associated at varying levels to hypoxia. These defined our core set, see Table 5. In addition to hypoxia gene sets these core gene sets contained Vascular endothelial growth factor (VEGF) gene which is generally up-regulated by hypoxic conditions and promotes normal blood vessel formation and angiogenesis related to tumor growth. In addition, hypoxia up-regulates the von Hippel-Lindau tumor suppressor gene (VHL) which plays a key role in VHL-hypoxia-inducible factor (VHL-HIF) pathway [22].

**Table 5:** Results for the Hypoxia gene sets in the Hypoxia/normal data set.

		**RS**		**GLAPA**			**GSEA**	
				
**Pathway**	**Size**	**Rank**	**P-value**	**Rank**	**P-value1**	**P-value2**	**Rank**	**P-value**
**MENSE_HYPOXIA_UP**	342	1	0.000	19	0.074	0.000	2	0.000
**HYPOXIA_FIBRO_UP**	51	3	0.000	18	0.081	0.004	9	0.000
**MENSE_HYPOXIA_DN**	14	31	0.000	1	0.054	0.000	4	0.000
**MANALO_HYPOXIA_UP**	305	11	0.001	30	0.083	0.000	7	0.004
**MENSE_HYPOXIA TRANSPORTER_GENES**	196	7	0.003	42	0.083	0.000	11	0.002
**MENSE_HYPOXIA APOPTOSIS_GENES**	38	10	0.000	31	0.086	0.000	26	0.014
**HYPOXIA_NORMAL_UP**	587	4	0.000	64	0.086	0.000	6	0.000
**HIF1_TARGETS**	98	8	0.004	66	0.088	0.006	37	0.013
**HYPOXIA_REVIEW**	220	5	0.002	116	0.111	0.005	8	0.000
**VEGFPATHWAY**	85	38	0.001	135	0.093	0.016	59	0.011
**HYPOXIA_REG_UP**	105	2	0.002	241	0.128	0.037	29	0.010
MANALO_HYPOXIA_DN	211	133	0.178	253	0.125	0.032	126	0.094
HIFPATHWAY	42	49	0.018	611	0.196	0.142	31	0.008
P53HYPOXIAPATHWAY	57	285	0.070	249	0.120	0.053	238	0.126
VHL_NORMAL_UP	1251	26	0.083	328	0.134	0.008	522	0.081
RCC_NL_UP	1529	15	0.033	239	0.116	0.001	727	0.222
VHL_RCC_UP	288	374	0.188	130	0.118	0.003	539	0.229
HYPOXIA_RCC_NOVHL_UP	159	528	0.201	177	0.111	0.014	560	0.260
HYPOXIA_RCC_UP	330	716	0.304	415	0.136	0.092	561	0.177

As Table 5 shows collectively the core set is strongly deregulated with respect to hypoxia. However we see greater variation in the median scores across the methods than in the case of P53. The median scores are 15, 130, and 31 for RS, GLAPA, GSEA respectively and these are all significant *p *< 0.001. As in the P53 case we ordered the gene sets according to the mean rank over the three methods in Table 5 and found the top eleven (in bold) to be highly ranked across all methods with median scores for this subset of 7, 42, and 9 for RS, GLAPA, and GSEA.

In summary there is still strong agreement across the three methods even though the variation in this data set is greater than that of the P53 example. We are not sure whether this is due to the much smaller sample size or greater biological variability in the induction of hypoxia. When we restrict ourselves to the nine highly ranked gene sets the variability is comparable to the P53 case.

#### Oncogenic pathways

In [10] five data sets were generated by activating the following five oncogenes Myc, Ras, E2F3, Src, and *β*-catenin in human primary mammary epithelial cell cultures. As a control GFP was also activated in these cell cultures. For each data set a signature of oncogenic deregulation was generated, for example a Myc, Ras, E2F3, Src, and *β*-catenin signatures. We took each signature and split them into up and down-regulated signatures based on whether the genes correlated with the Myc phenotype or the GFP phenotype.

We added these 10 gene sets to those in MSigDB. In this case the core gene sets for each data set are the corresponding two up and down regulated gene sets. For example, in the Ras data set we expect the up and down-regulated gene sets to rank towards the top.

We applied the three methods for measuring enrichment of the extended gene set database in these five data sets. The rank of the respective up/down gene set for each oncogenic perturbation is reported in Table 6. A complete description of the results obtained on these data sets is reported in Additional file 4, Additional file 5, Additional file 6, Additional file 7 and Additional file 8. In this case the three methods were not similar and GSEA seems to be much better at highlighting the respective pathway deregulation. We suspect the reason that GLAPA does not rank the deregulated pathway as strongly as GSEA is that in these oncogenic perturbations a multitude of pathways are deregulated. For example in the Ras data set the cross-validation prediction error for the two Ras gene sets are *e *= 0.0 with very small p-values (p-values .007 and .004 for Ras up and down). However, GLAPA measured an error rate of 0.0 for 70% of the gene sets and these estimates also had very small p-values, < 0.01. This situation also occurs in the other data sets. This suggests that perturbation of the oncogenes results in deregulation across many pathways and deep functional changes.

**Table 6:** Deregulation of the five oncogenes as measured by the three methods.

		**RS**		**GLAPA**			**GSEA**	
				
**Pathway**	**Size**	**Rank**	**P-value**	**Rank**	**P-value1**	**P-value2**	**Rank**	**P-value**
Myc_up	119	60	0.083	997	0.005	0.831	7	0.000
Myc_down	129	1099	0.629	242	0.006	0.199	6	0.000

Ras_up	195	1181	0.726	842	0.007	0.998	6	0.019
Ras_down	153	439	0.442	1216	0.004	0.991	5	0.006

E2F3_up	138	35	0.088	79	0.012	0.472	4	0.000
E2F3_down	160	994	0.619	1111	0.016	0.965	10	0.008

Src_up	28	182	0.186	1409	0.018	0.513	17	0.060
Src_down	45	41	0.104	781	0.019	0.303	9	0.032

*β*-catenin_up	43	231	0.198	1588	0.063	0.952	4	0.006
*β*-catenin_down	55	87	0.105	495	0.016	0.211	6	0.011

The point of this example is that when the difference between the two phenotypes is extensive and characterized by a wide variety of pathways or gene sets, GLAPA and RS may not be able to focus on the most deregulated pathways while GSEA, at least in this example, finds these gene sets.

#### Breast cancer

The deregulation of the whole MSigDB collection was measured in the breast cancer data set composed of patients with recurrent and non recurrent phenotypes [11]. We compared the three methods in detecting deregulation of some pathways related to these phenotypes. The first gene set we considered was the P53 pathway. This pathway is in general altered in many types of cancers [1] and its importance as a marker for recurrence in breast cancer is well known [23]. GLAPA detected a strong deregulation of P53_BRCA1_UP pathway (rank = 2, P-value1 = 0.009, P-value2 = 0.001) and this finding was confirmed by RS (rank = 8, P-value = 0.002).

A further analysis concerned the cell cycle deregulation. This pathway has been identified as one of the hallmarks of cancer [24] and, more important, an increased activity of the cell cycle has been linked to more aggressive tumors [25]. GSEA was the only method which highlighted the deep alteration of CELL_CYCLE_CHECKPOINT pathway (rank = 8, P-value = 0.010) in this data set. GLAPA only weakly confirmed such deregulation (rank = 170, P-value1 = 0.07, P-value2 = 0.08).

Finally, we analyzed pathways involving E2F transcription factors which play a key role in tumor progression and in particular in breast cancer [25]. In fact, alterations in E2Fs increase cell proliferation and render cells insensitive to antigrowth signals [24]. RS and GSEA revealed significant deregulations of E2F3 (rank = 32, P-value = 0.014) and REN_E2F1_TARGETS (rank = 54, P-value = 0.031) signatures respectively, while GLAPA confirmed only weakly the result of RS (rank = 136, P-value1 = 0.063, P-value2 = 0.136).

#### Lung cancer

We compared the three methods in NSCLC data set of patients with adenocarcinoma and squamous phenotypes [12]. To this end, we measured the alteration of Myc oncogene in this data set. The Myc oncogene family encodes a group of nuclear phosphoproteins that plays a role in cell growth and in the development of human tumors. In particular, overexpression and amplification of Myc family members have been reported in the majority of Small Cell Lung Cancer (SCLC) and in a subset of Non-Small Cell Lung Cancers (NSCLC) [26]. GLAPA was able to detect a strong deregulation of the Myc signature (rank = 5, p-value1 < 10^-3^, p-value2 = 0.008) and this evidence was confirmed by RS (rank = 80, p-value = 0.029). Also GSEA detected a deep deregulation of this oncogene, highlighting a different signature of this gene (YEN_MYC_WT, rank = 21, p-value = 0.016).

Previous work has linked Ras activation with the development of adenocarcinomas of the lung [10]. RS and GLAPA shown similar abilities in highlighting Ras deregulation in this data set providing significant ranks of 51 (p-value = 0.03) and 61 (p-value1 < 0.001, p-value2 = 0.089) respectively.

Finally, we measured alterations of cell cycle pathway which is known to be involved in NSCLC [27]. RS and GSEA detected cell cycle alterations in the current experimental conditions. In fact, RS highlighted SERUM_FIBROBLAST_CELLCYCLE (rank = 7, p-value = 0.018) and GSEA detected CELL_CYCLE_REGULATOR (rank = 1, p-value1, p-value2 < 10^-3^). These findings were only weakly confirmed by GLAPA. In fact, in the first case GLAPA reported (rank = 317, p-value1 < 0.001, p-value2 = 0.472) and in the second one reported (rank = 178, p-value1 < 0.001, p-value2 = 0.060).

### Variation in rankings across methods

**Figure 1:**
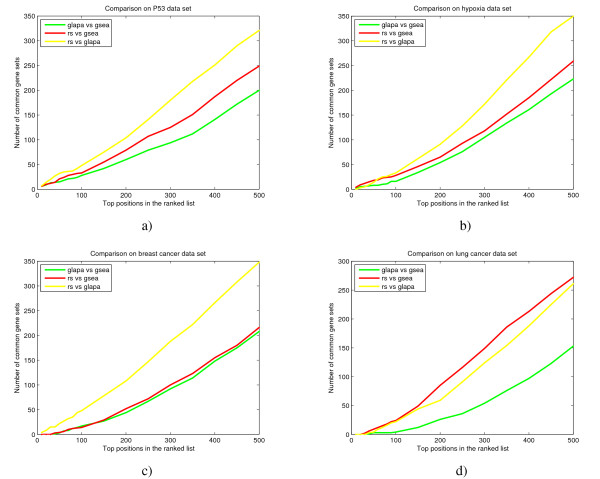
**Overlaps of the ranks of gene sets across the three methods in a) P53, b) hypoxia, c) breast cancer and d) lung cancer data sets**. *x*-axis represents the number of top gene sets considered and *y*-axis represents the overlap in each pairwise comparison.

To further quantify the similarity of the enrichment estimates across the three methods we compare the overlaps of the ranks of gene sets across the three methods. These comparisons are made pairwise. For each pair of methods for example GSEA versus GLAPA we compute the overlap of the two rank ordered gene sets as a function of the number of gene sets considered. In the four plots in Figure 1 the x-axis is the number of top gene sets considered and the y-axis is the overlap. This is displayed for the P53, hypoxia, beast cancer, and lung cancer data in Figures 1(a, b, c, d). The different pairwise comparisons are displayed in different colors for the three pairwise comparisons. From this picture it is obvious that there is a greater similarity between RS and GLAPA in evaluating pathway deregulation and this similarity is uniform across examples. For example, among the top 250 enriched gene sets in the P53 example the overlap between RS and GLAPA is 60% (p-value = 0 by Fisher's exact test) of gene sets in common, while this number reduces to 30% (p-value = 0) comparing GLAPA with GSEA.

In summary the rankings overlap significantly across the three methods but the similarity between GLAPA and RS is considerably greater.

### Associative versus predictive scores

In this subsection we focus on GLAPA versus RS. Although these two methods provide similar rankings the statistic computed and therefore the significance of this statistic are different. In the case of GLAPA the statistic, the cross-validation error, is predictive - how well do the genes in the gene set predict the phenotype of interest, for example hypoxic condition. In RS setting is that of classical two sample hypothesis testing where we measure a set of means and ask if these means are different under the null hypothesis that the two conditions or phenotypes are identical. The predictive statistic or requirement is much more stringent than the associative case. The following simple example illustrates this: consider a pathway composed of a single gene *x *and suppose that the distribution of expression levels of this gene is *x*_*I *_~ No(0, 1) in phenotype I (control) and *x*_*II *_~ No(*ε*, 1) in phenotype II (case) with *ε *> 0 arbitrarily small. Given enough observations a two sample t-test or any other reasonable hypothesis test will provide strong evidence for rejecting the null hypothesis - these two phenotypes have the same means. However, the classification accuracy of any classifier, even the optimal Bayes classifier will be arbitrarily close to 50%. This phenomenon is not just theoretical but we see this in our analyses of the various data sets. To highlight this we examined the overlap of significant gene sets obtained by GLAPA and RS in three of the examples, P53, breast cancer, and lung cancer. We did not include hypoxia due its the small sample size. In the case of RS significant gene sets were those with p-values less than 0.05 and in the case of GLAPA both p-values were required to be less than 0.05. We consider the gene sets found significant by GLAPA to be predictive and the ones found significant by RS associative. Table 7 lists the number of significant gene sets via both methods and their overlap. The overlap between the methods is substantial and significant by Fisher's exact test. See Additional file 9, Additional file 10 and Additional file 11 for this list of gene sets. An interesting example of a gene set that is found to predictive in addition to being associative by GLAPA and RS respectively is the P53 pathway in breast cancer. This suggests that this pathway is predictive of recurrence and the effect size of the deregulation measured by the associative test is large. This would be an important pathway to further study. Another example of this is the case of alterations of cell cycle pathways that we report in the lung cancer section where pathways were detected by RS and GSEA but failed the second p-value test of GLAPA suggesting that they are weakly predictive.

**Table 7:** Number of statistical significant gene sets highlighted by RS with p-value < 0.05 and by GLAPA with p-value1, p-value2 < 0.05.

**Dataset**	**RS**	**GLAPA**	**Common gene sets**
P53	91	35	27
Breast	77	47	27
Lung	340	76	31

## Discussion and conclusion

Many methods have been developed in the last few years to assess the differential enrichment of sets of genes [2-9] highlighting the importance of pathway analysis in the study of complex diseases, and, in particular, in oncology. In this paper we have compared four of these techniques which belong to two different classes of methods. Fisher's exact test [3], GSEA [2], RS [8,9] are associative methods which quantify the deregulation of a gene set comparing the distributions of the expression levels of the genes in the gene set in the two phenotypic conditions analyzed. GLAPA [7] is a predictive method which measures deregulation by assessing the prediction accuracy of the phenotype of new subjects by using the expression levels of the genes in the gene set. The performances of these methods as well as their intrinsic properties have been highlighted and characterized by analyzing the methods in different experimental conditions. Numerous aspects have emerged by our comparative study. Concerning the methods analyzed, the simulation studies confirm that Fisher's exact test is considerably worse than the other three methods as it is unable to detect gene sets with modest deregulation. On the contrary, RS and GSEA are able to highlight subtle alterations. The former does not suffer of the simultaneous presence of up and down regulated genes in the gene set, while the latter is able to detect the true deregulation even whether, as in the case of oncogenic pathways, the phenotypic distinction is characterized by a wide variety of altered pathways. Although the performances of these two approaches are comparable, GSEA does come with easy to use code and a graphical interface as well as a compendium of gene sets which in many respects trumps statistical rigor. GLAPA deserves a separate discussion as it assesses deregulation through a predictive statistics. We have made explicit the deep difference existing between associative and predictive statistics. This method is more conservative and is able to detect deregulation when the difference between the two phenotypic conditions is marked. Such property has been confirmed by the analysis of the method on breast and lung cancer data sets in which GLAPA revealed the alteration of pathways and oncogenes relevant for these pathologies.

Concerning the gene sets adopted in our study, we have shown that using core sets, composed of different signatures of the same gene or pathways thought to be correlated in the data set, makes the analysis less sensitive to the noise embedded in the data. The reason for considering core sets is that gene sets are constructed under a variety of contexts and conditions and looking at a group of sets helps average out this variation. This aspect is evident in P53 and hypoxia data sets.

The purpose of our comparative study was to provide suggestions for users of gene set methods regarding which method to use under which condition. The results do not allow to determine univocally the most suitable method as one method does not always outperform the others. However, we can make some general recommendations. In terms of significance and the type of statistic used, GSEA and RS are more similar and provide comparable information. In this context if there are no computational constraints we suggest the use of GSEA especially if one suspects that the data consists of many deregulated pathways as was the case in oncogenic perturbation example. We recommend running both GSEA and GLAPA or RS and GLAPA in tandem as they provide complementary information. In the case of developing drug targets or when it is important to have a measure of the predictive accuracy on individuals rather than global differences in distributions between the two phenotypes GLAPA is well suited. Also of fundamental importance in all these methods is which gene sets one is using and also the consideration of splitting gene sets into up and down regulated subsets. This was seen in the P53 example and also is the case in the oncogenic perturbation example. We also suggest that users of these methods look carefully at the outcomes of these enrichment studies and realize that variation in significance across methods often is reflective of biological variation in that there may be many underlying pathways or sets of genes that are differentially expressed in the data set.

## Authors' contributions

NA and SM conceived the study. LA, RM, TMC and AD'A designed the algorithms and conduced the experiments and, together with ADi, SM and NA, they evaluated and compared the experimental results. All authors read and approved the final manuscript.

## Conflict of interests

The authors declare that they have no competing interests.

## Supplementary Material

Additional file 1 **Supplement of simulations**. Comparison of the methods in different simulation scenarios. Click here for file

Additional file 2 **Complete results in P53 data set**. Complete results obtained in P53 data set. Click here for file

Additional file 3 **Complete results in hypoxia data set**. Complete results obtained in Hypoxia data set. Click here for file

Additional file 4 **Complete results in bcat data set**. Complete results obtained in *α*-catenin data set. Click here for file

Additional file 5 **Complete results in e2f3 data set**. Complete results obtained in E2F3 data set. Click here for file

Additional file 6 **Complete results in myc data set**. Complete results obtained in Myc data set. Click here for file

Additional file 7 **Complete results in ras data set**. Complete results obtained in Ras data set. Click here for file

Additional file 8 **Complete results in src data set**. Complete results obtained in Src data set. Click here for file

Additional file 9 **Significant gene sets in P53 data set**. Significant gene sets highlighted by RS and GLAPA in P53 data set. Click here for file

Additional file 10 **Significant gene sets in breast data set**. Significant gene sets highlighted by RS and GLAPA in breast cancer data set. Click here for file

Additional file 11 **Significant gene sets in lung data set**. Significant gene sets highlighted by RS and GLAPA in lung cancer data set. Click here for file
