# Supplementary material for: Comparative study of gene set enrichment methods
Source: BMC Bioinformatics. 2009 Sep 2;10:275. doi: 10.1186/1471-2105-10-275 (PMC2746222; doi:10.1186/1471-2105-10-275)
Supplement: Additional file 1 — Supplement of simulations. Comparison of the methods in different simulation scenarios. [file 1471-2105-10-275-S1.doc]

# Supplemental material

We analyzed the performances of the RS, GLAPA and GSEA methods under two different hypotheses of gene expression level distribution. In the first case, the measurements were generated as a Student *t* distribution $t\left(\nu\right)$ with a number of degrees of freedom $\nu=3$. The displacements in the five different scenarios were in units of standard deviation given by $\sigma=\sqrt{\frac{\nu}{\nu-2}}$. The mean and standard error of the p-values computed over the 20 simulations are reported in Table S1. Note that the relative performances of the three methods remain the same under the hypothesis of *t*-distributed gene expression levels.

|  | RS | | GLAPA | | | | GSEA | |
| --- | --- | --- | --- | --- | --- | --- | --- | --- |
|  | P-value | | P-value1 | | P-value2 | | P-value | |
|  | mean | se | mean | se | mean | se | mean | se |
| 1 | 0.0233 | 0.0140 | 0.0920 | 0.0172 | 0.0312 | 0.0096 | 0.0006 | 0.0003 |
| 2 | 0.0027 | 0.0019 | 0.0296 | 0.0078 | 0.0023 | 0.0011 | 0.0002 | 0.0001 |
| 3 | 0.0092 | 0.0067 | 0.0198 | 0.0075 | 0.0028 | 0.0021 | 0.0007 | 0.0004 |
| 4 | 0.0103 | 0.0041 | 0.0042 | 0.0019 | 0.0002 | 0.0001 | 0.0044 | 0.0017 |
| 5 | 0 | 0 | 0.0007 | 0.0004 | 0 | 0 | 0.0836 | 0.0136 |

**Table S1**. Results of simulation study: comparison of RS, GSEA, and GLAPA. P-values for the first gene set for the three methods (columns) and five different scenarios (rows) described in the text in the case of the expression levels of genes in a gene set are distributed according to a *t*-distribution with 3 degrees of freedom.

We compared the performances of the three methods under the hypothesis of dependency of genes in gene sets. In particular, we carried out a simulation study in which the expression levels of the genes in each gene set were generated according to a No(0,Σ) where Σ, the covariance matrix of the genes in the gene set, is a 20×20 matrix with entries σ_ij_ given by:

$$\sigma_{ij}=\left\{\begin{matrix} 1 \\ \rho\end{matrix}\begin{matrix} \\ \end{matrix}\begin{matrix} if \\ if \end{matrix}\begin{matrix} \\ \end{matrix}\begin{matrix} i=i \\ i\neq j \end{matrix}\right.$$

with ρ=0.5. We considered the same first 4 scenarios as in the main text and the simulation results are reported in table S2. The fifth scenario is not included since the covariance structure proposed cannot be consistent ten genes being up-regulated and ten genes being down regulated. The hypothesis of dependency of genes in gene sets does not modify the performances of RS and GSEA but, on the contrary, increases the capability of GLAPA to detect the deregulated gene set.

|  | RS | | GLAPA | | | | GSEA | |
| --- | --- | --- | --- | --- | --- | --- | --- | --- |
|  | P-value | | P-value1 | | P-value2 | | P-value | |
|  | mean | se | mean | se | mean | se | mean | se |
| 1 | 0.0016 | 0.0008 | 0.0264 | 0.0061 | 0.0198 | 0.0038 | 0.0009 | 0.0003 |
| 2 | 0.0018 | 0.0005 | 0.0001 | 0.0001 | 0 | 0 | 0.0122 | 0.0101 |
| 3 | 0.0206 | 0.0027 | 0 | 0 | 0 | 0 | 0.0032 | 0.0008 |
| 4 | 0.1027 | 0.0126 | 0 | 0 | 0 | 0 | 0.0281 | 0.0107 |

**Table S2**. Results of simulation study: comparison of RS, GSEA, and GLAPA. P-values for the first gene set for the three methods (columns) and four different scenarios (rows) described in the text in the case of the expression levels of genes in a gene set are distributed according to a No(0,Σ).
